# Supplementary material for: Mapping First In Vivo Ebolavirus Infection Events
Source: bioRxiv. 2026 Sep 18:2026.09.16.752249. Preprint. [Version 1] doi: 10.64898/2026.09.16.752249 (PMC13596410; doi:10.64898/2026.09.16.752249)

bioRxiv preprint doi: https://doi.org/10.64898/2026.09.16.752249; this version posted September 18, 2026. The copyright holder for this preprint (which was not certified by peer review) is the author/funder, who has granted bioRxiv a license to display the preprint in perpetuity. It is made available under a CC-BY 4.0 International license.

Supplemental Figure 1. Luciferase Activity in Skin-draining Inguinal Lymph Nodes. A) Representative bioluminescence images of the draining inguinal lymph nodes on day 7. Following euthanasia, the abdominal skin was opened to expose the inguinal lymph nodes, which were imaged using IVIS. Red circles indicate the regions of interest (ROIs) used for quantification of luciferase activity. **B)** Quantification of luciferase activity in the draining inguinal lymph nodes. Detectable luciferase signals were observed in two individual mice, with no statistically significant differences among treatment groups. One mouse in the concentrated trVLP group died on day 5 and was therefore not included in the day 7 lymph-node imaging.

Supplemental Figure 2. **Representative Flow Cytometry Gating for Skin Samples**. Representative gating strategy for analysis of GFP-positive immune-cell populations in skin. Following exclusion of debris, doublets, and nonviable cells, $CD45^+$ immune cells were identified and sequentially gated based on CD11c, MHC-II, CD207, and GFP expression. Representative GFP plots from mice receiving PBS, unconcentrated trVLP-containing supernatant, or concentrated trVLPs are shown.

Supplemental Figure 3. Representative Flow Cytometry Gating for Draining Lymph Node Samples. Representative flow cytometry plots of draining inguinal lymph node cells from mice receiving PBS, unconcentrated trVLP-containing supernatant, or concentrated trVLPs. The gating strategy was performed as described in Supplemental Figure 2.

Supplemental Figure 4. Original silver-stained SDS-PAGE gels of EBOV trVLP preparations. Original and representative silver-stained SDS-PAGE gel images used for characterization of the indicated trVLP preparations. Molecular-weight markers are shown

bioRxiv preprint doi: https://doi.org/10.64898/2026.09.16.752249; this version posted September 18, 2026. The copyright holder for this preprint (which was not certified by peer review) is the author/funder, who has granted bioRxiv a license to display the preprint in perpetuity. It is made available under a CC-BY 4.0 International license.

in kilodaltons (kDa). The approximate migration positions of GP, VP40, and VP24 are indicated. The uncropped gel images are provided to show the overall protein profiles of the analyzed preparations**.**

Supplemental Figure 5. Original Western blot images of EBOV trVLP proteins. Original membrane and Western blot images used for characterization of the indicated trVLP preparations. The membrane image prior to sectioning and the corresponding immunoblots probed for individual EBOV proteins are shown. The positions of GP, VP40, VP24, L, NP, and VP35 are indicated.

bioRxiv preprint doi: https://doi.org/10.64898/2026.09.16.752249; this version posted September 18, 2026. The copyright holder for this preprint (which was not certified by peer review) is the author/funder, who has granted bioRxiv a license to display the preprint in perpetuity. It is made available under a CC-BY 4.0 International license.

Liu *et al.* Supplemental Fig. 1

A

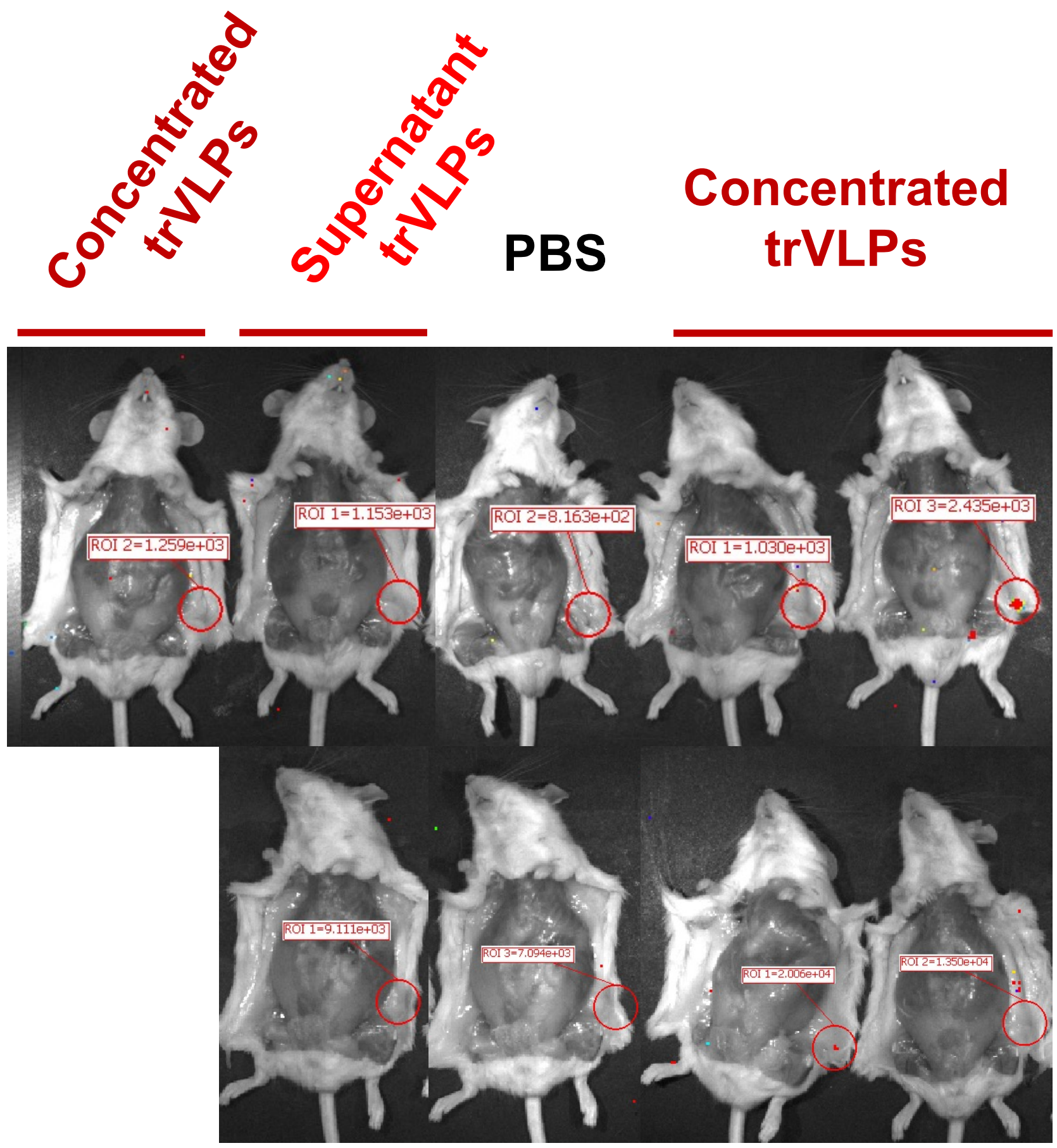

B

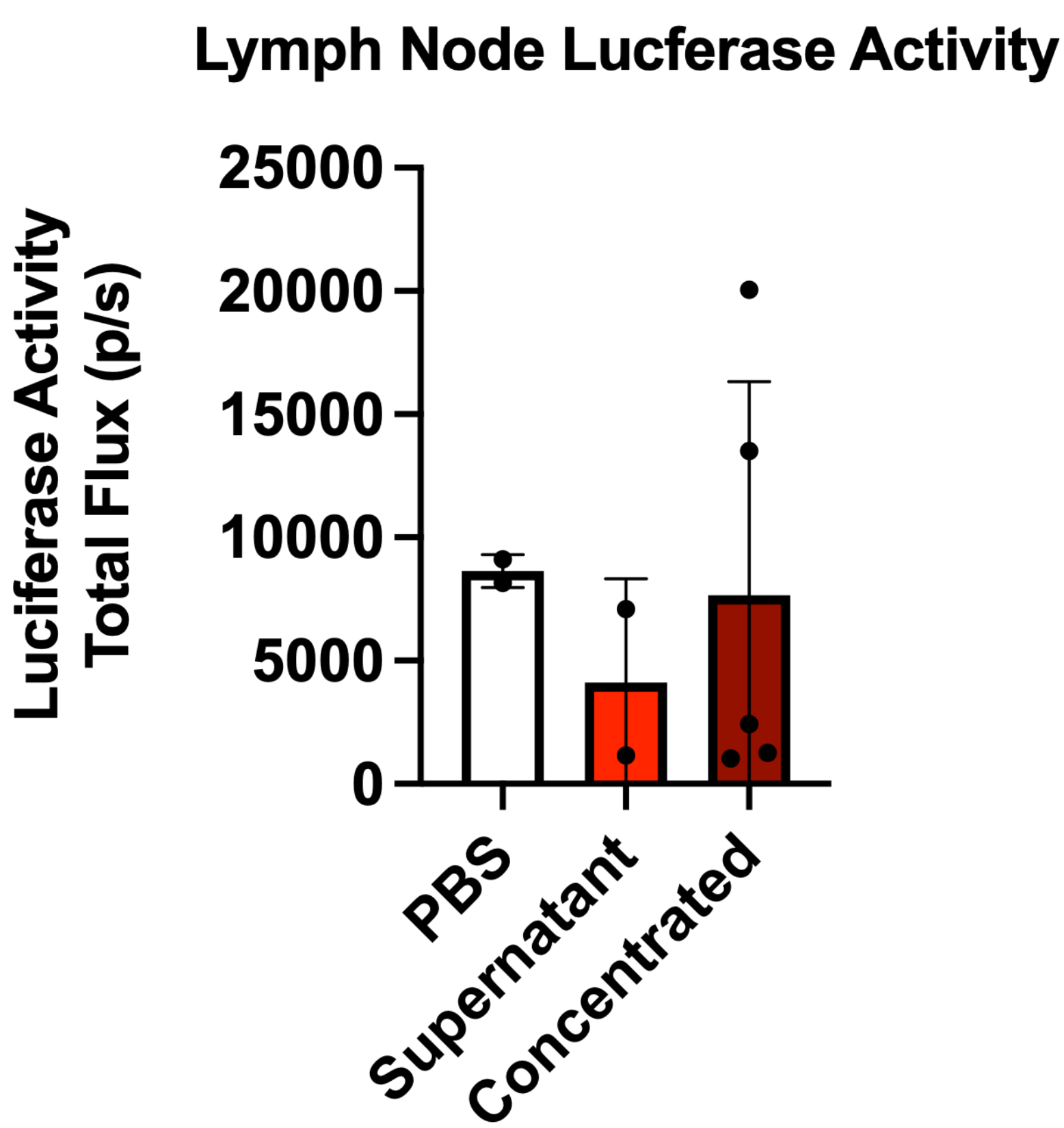

bioRxiv preprint doi: https://doi.org/10.64898/2026.09.16.752249; this version posted September 18, 2026. The copyright holder for this preprint (which was not certified by peer review) is the author/funder, who has granted bioRxiv a license to display the preprint in perpetuity. It is made available under a CC-BY 4.0 International license.

Liu *et al.* Supplemental Fig. 2

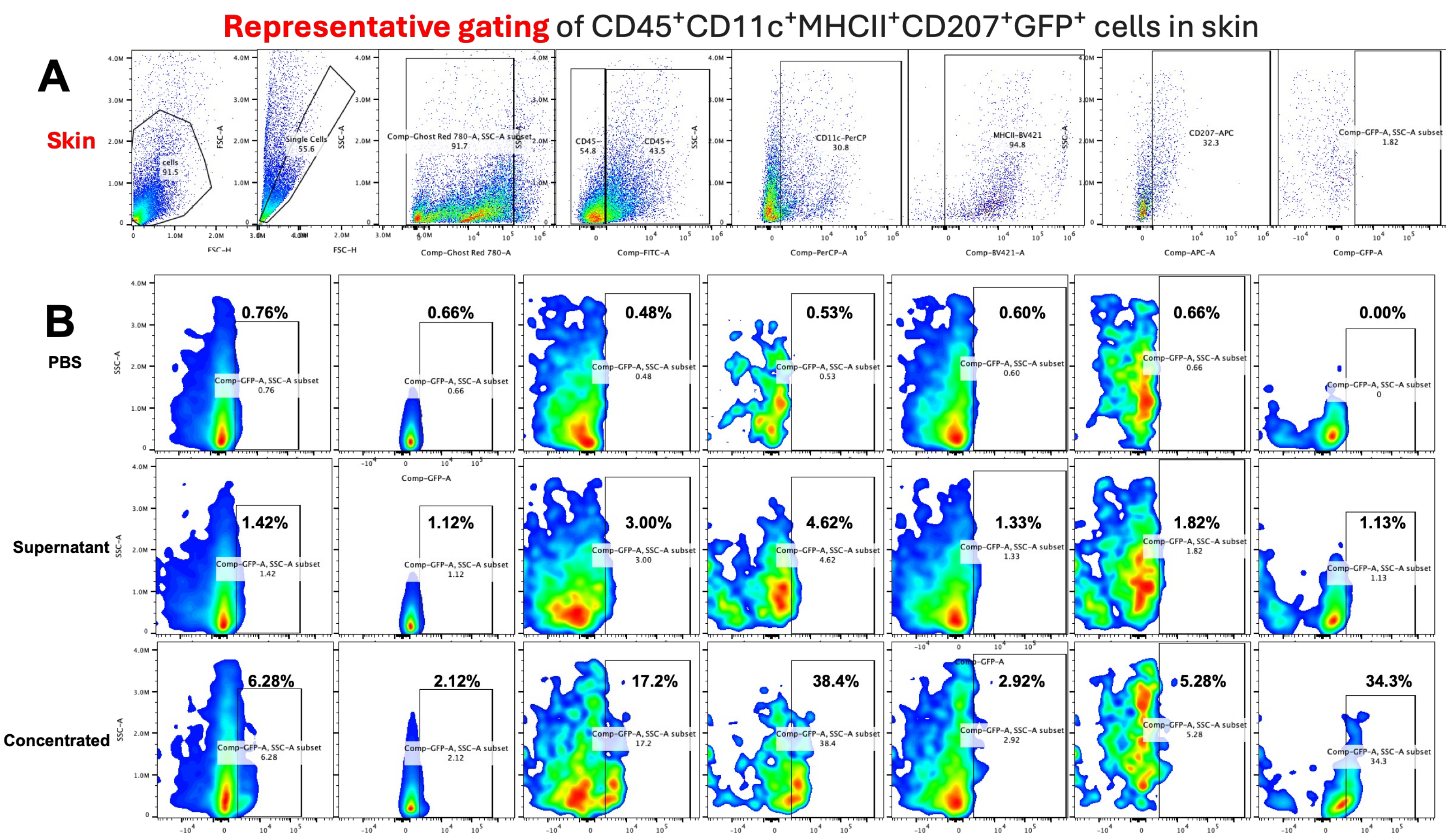

bioRxiv preprint doi: https://doi.org/10.64898/2026.09.16.752249; this version posted September 18, 2026. The copyright holder for this preprint (which was not certified by peer review) is the author/funder, who has granted bioRxiv a license to display the preprint in perpetuity. It is made available under a CC-BY 4.0 International license.

Liu *et al.* Supplemental Fig. 3

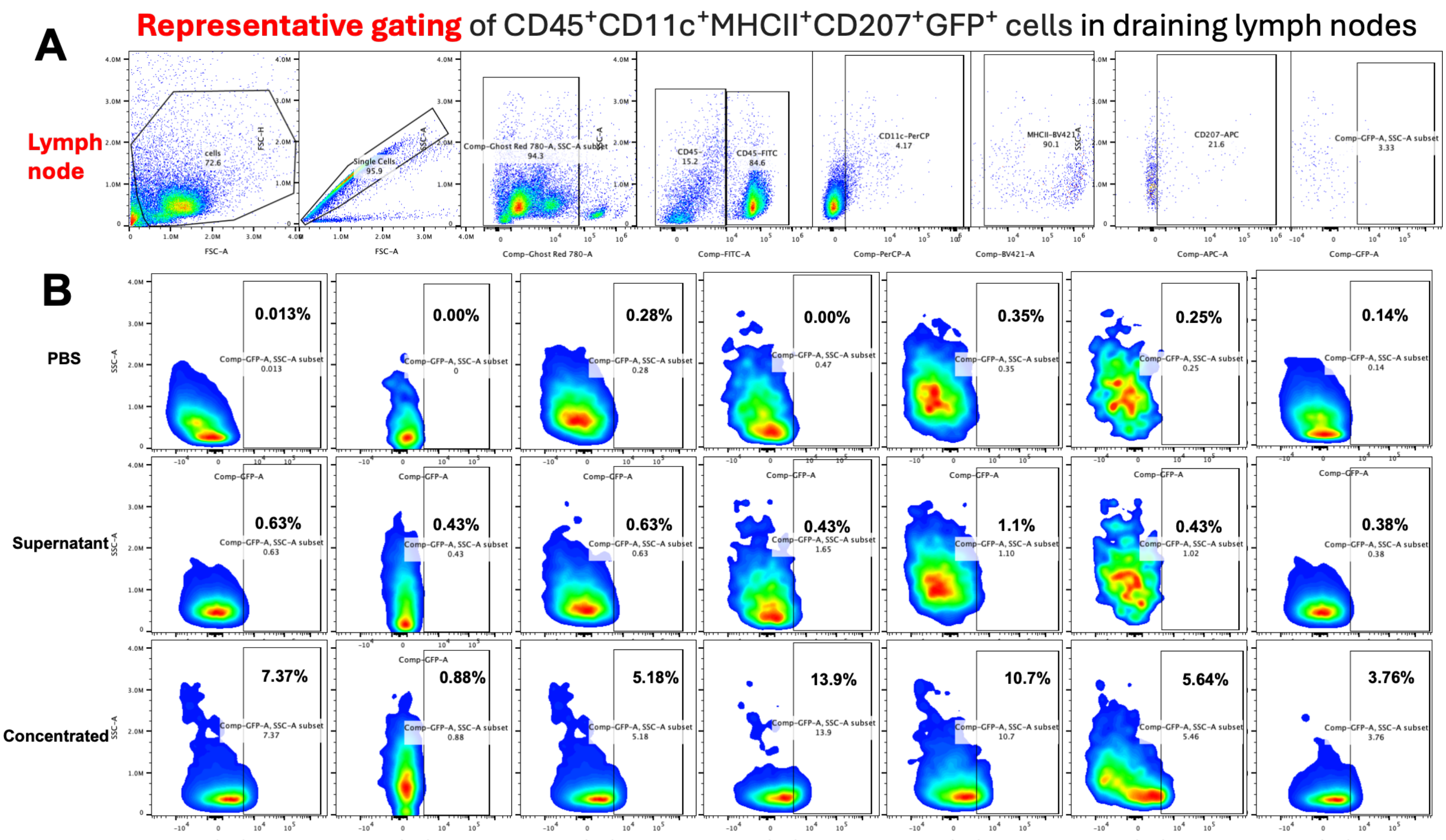

bioRxiv preprint doi: https://doi.org/10.64898/2026.09.16.752249; this version posted September 18, 2026. The copyright holder for this preprint (which was not certified by peer review) is the author/funder, who has granted bioRxiv a license to display the preprint in perpetuity. It is made available under a CC-BY 4.0 International license.

Liu *et al.* Supplemental Fig. 4

A

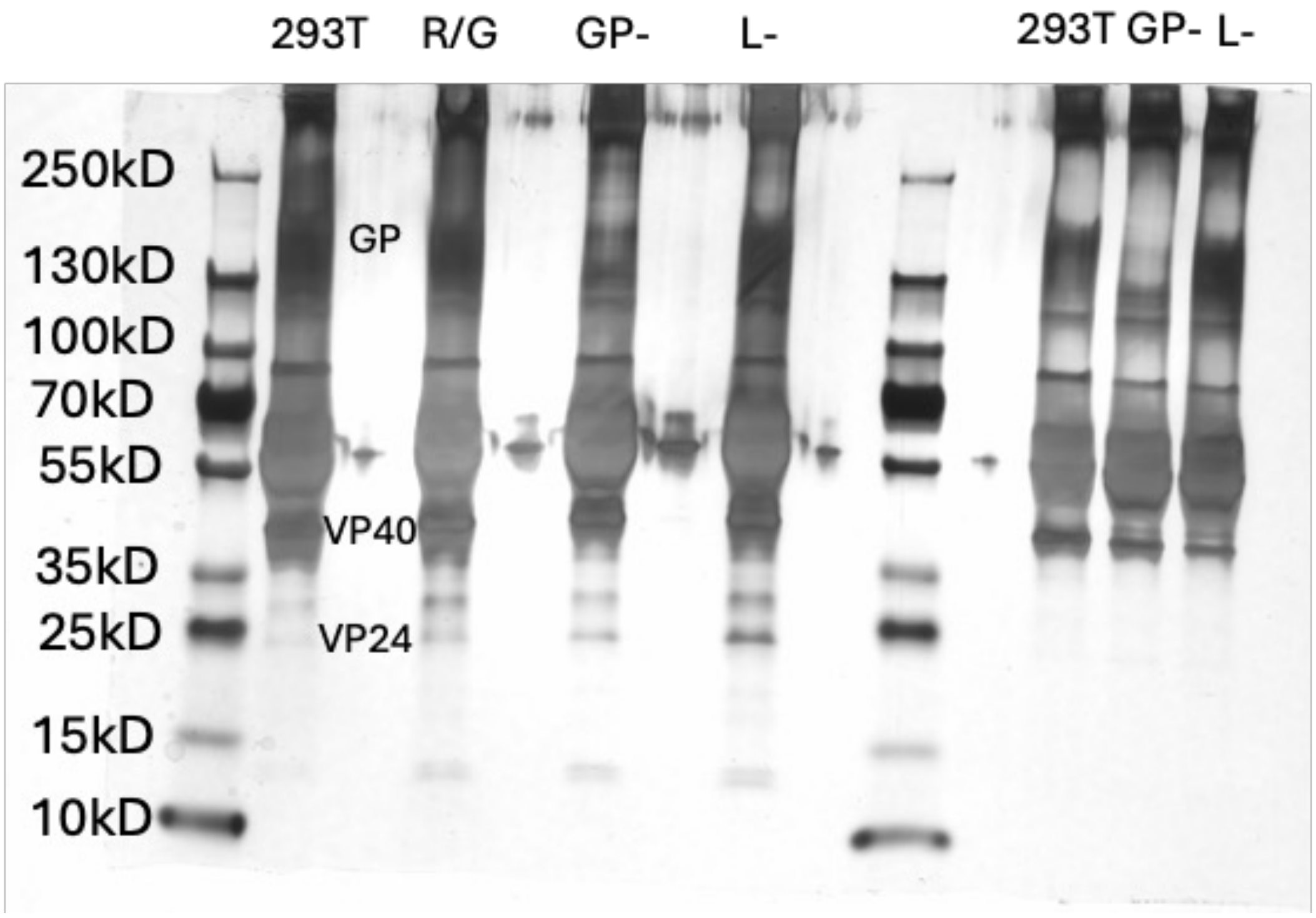

B

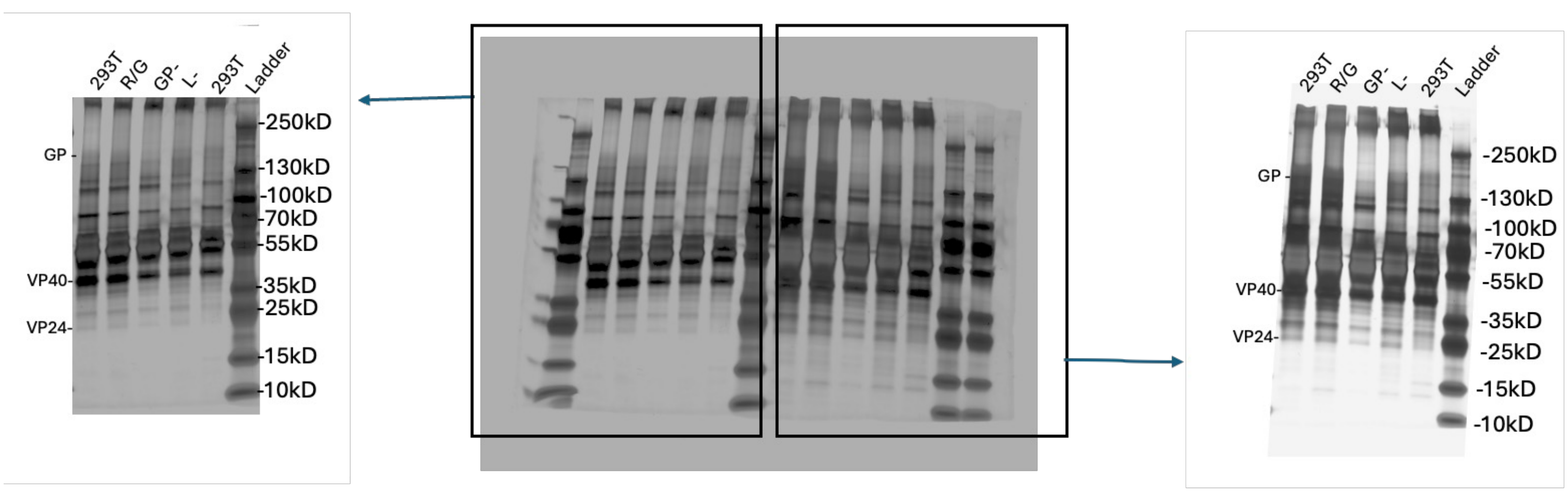

bioRxiv preprint doi: https://doi.org/10.64898/2026.09.16.752249; this version posted September 18, 2026. The copyright holder for this preprint (which was not certified by peer review) is the author/funder, who has granted bioRxiv a license to display the preprint in perpetuity. It is made available under a CC-BY 4.0 International license.

Liu *et al.* Supplemental Fig. 5

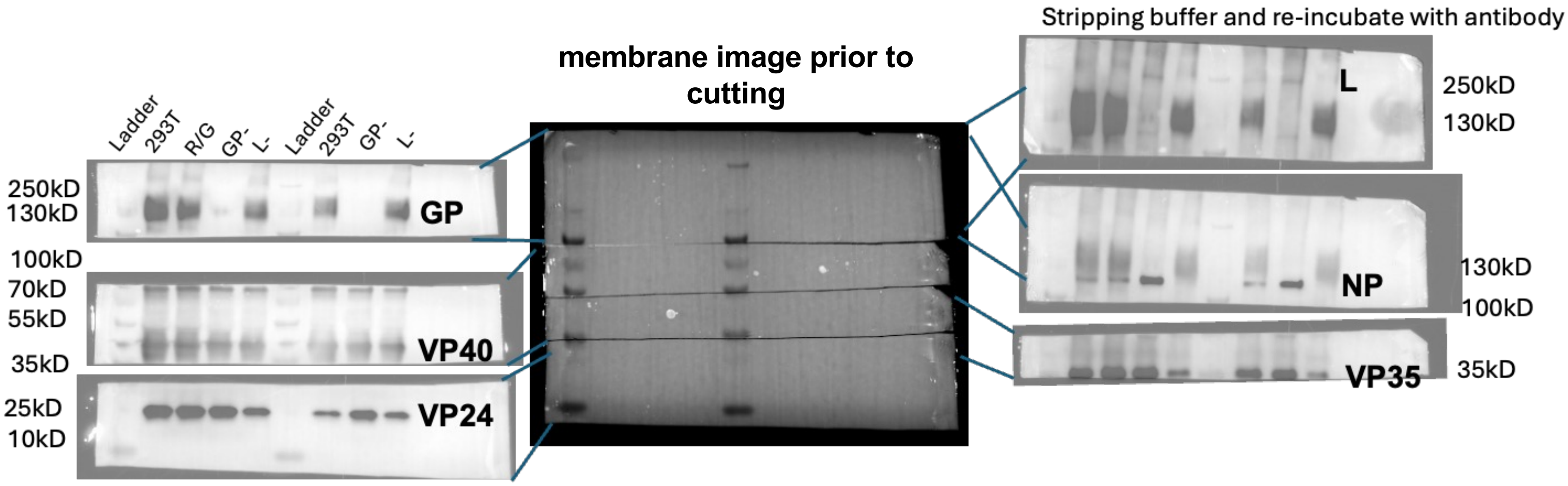

Supplement: Supplementary file 1 [file NIHPP2026.09.16.752249v1-supplement-1.pdf]
